# Effectiveness of early switching from intravenous to oral antibiotic therapy in *Staphylococcus aureus* prosthetic bone and joint or orthopedic metalware-associated infections

**DOI:** 10.1186/s12891-021-04191-y

**Published:** 2021-03-30

**Authors:** Hélène Boclé, Jean-Philippe Lavigne, Nicolas Cellier, Julien Crouzet, Pascal Kouyoumdjian, Albert Sotto, Paul Loubet

**Affiliations:** 1 Department of Infectious and Tropical Diseases, CHU Nîmes, University of Montpellier, Nîmes, France; 2 Virulence Bactérienne et Maladies Infectieuses, Inserm U1407, Université de Montpellier, Nîmes, France; 3 Department of Microbiology and Hospital Hygiene, CHU Nîmes, University of Montpellier, Nîmes, France; 4 Department of Orthopedic and Trauma Surgery, CHU Nîmes, University of Montpellier, Nîmes, France

**Keywords:** *Staphylococcus aureus*, Prosthetic bone and joint infections, Oral antibiotic treatment, Intravenous antibiotic treatment

## Abstract

**Background:**

The optimal duration of intravenous antibiotic therapy in *Staphylococcus aureus* prosthetic bone and joint infection has not been established. The objective of this study was to compare the effect of early and late intravenous-to-oral antibiotic switch on treatment failure.

**Patients and methods:**

We retrospectively analyzed all adult cases of *S. aureus* prosthetic bone and joint or orthopedic metalware-associated infection between January 2008 and December 2015 in a French university hospital. The primary outcome was treatment failure defined as the recurrence of *S. aureus* prosthetic bone and joint or orthopedic metalware-associated infection at any time during or after the first line of medical and surgical treatment within 2 years of follow-up. A Cox model was created to assess risk factors for treatment failure.

**Results:**

Among the 140 patients included, mean age was 60.4 years (SD 20.2), and 66% were male (*n* = 92). Most infections were due to methicillin-susceptible *S. aureus* (*n* = 113, 81%). The mean duration of intravenous antibiotic treatment was 4.1 days (SD 4.6). The majority of patients (119, 85%) had ≤5 days of intravenous therapy. Twelve patients (8.5%) experienced treatment failure. Methicillin-resistant *S. aureus* infections (HR 11.1; 95% CI 1.5–111.1; *p* = 0.02), obesity (BMI > 30 kg/m^2^) (HR 6.9; 95% CI1.4–34.4, *p* = 0.02) and non-conventional empiric antibiotic therapy (HR 7.1; 95% CI 1.8–25.2; *p* = 0.005) were significantly associated with treatment failure, whereas duration of intravenous antibiotic therapy (≤ 5 or > 5 days) was not.

**Conclusion:**

There was a low treatment failure rate in patients with *S. aureus* prosthetic bone and joint or orthopedic metalware-associated infection with early oral switch from intravenous to oral antibiotic therapy.

## Introduction

Despite considerable progress on their prevention, prosthetic bone and joint or orthopedic metalware-associated infections remain a major public health problem in terms of cost, morbidity and mortality [1, 2]. *Staphylococcus aureus* is the pathogen most frequently involved in prosthetic joint infections, responsible for more than 50% of documented infections [3].

The management of prosthetic bone and joint or orthopedic metalware-associated infection is complex, requiring coordination across multiple specialties and is limited by a lack of standardized diagnostic criteria and recommendations such as for the duration of intravenous treatment. The Infectious Diseases Society of America (IDSA) recommends that *S. aureus* infections are treated, regardless of the surgical intervention, with 2 to 6 weeks (6 weeks if rifampicin is not possible) of intravenous antistaphylococcal antibiotic therapy combined with oral rifampicin followed by oral antibiotic therapy for 3 to 6 months, depending on the affected joint [4]. The French Infectious Diseases Society (SPILF) [5] recommends 2 weeks of intravenous treatment and a total duration of 6 to 12 weeks, but acknowledges that the duration of parenteral antibiotic therapy has not been validated, and proposes an earlier oral switch if possible, according to “expert opinion”.

Based on the long-standing experience in our center of early switching from intravenous to oral antibiotic in prosthetic bone and joint or orthopedic metalware-associated infection, we wished to evaluate the impact of the duration of intravenous antibiotic treatment in *S. aureus* prosthetic bone and joint or orthopedic metalware-associated infection on outcomes after 2 years for duration of hospitalization and occurrence of vascular catheter-related infections, as well as factors associated with failure of the early oral treatment.

## Patients and methods

### Study design and participants

We performed a single center, retrospective, observational study including all patients with a first episode of monomicrobial *S. aureus* prosthetic bone and joint or orthopedic metalware-associated infection who underwent appropriate surgical management, between January 1, 2008 to December 31, 2015 in all departments of our University Hospital. Adult patients with *S. aureus* positive samples were identified from the Microbiology Laboratory database (GLIMS®, MIPS, Gent, Belgium). Those with non-bone and joint infection or non- prosthetic bone and joint or orthopedic metalware-associated infection were excluded. As were those with polymicrobial or bacteriologically unproven infection, with unconventional treatment i.e. suppressive antibiotic therapy (defined as the indefinite administration of antibiotics without removing the prosthesis to reduce symptoms and/or prevent infection progression), therapeutic abstention (defined as the absence of medical and/or surgical management due to patient medical history and underlying conditions) or inappropriate surgical management according to the international recommendations [4, 5] as we considered that they were at increased risk of treatment failure due to non-optimal care.

### Assessment and endpoints

Age, Charlson score [6], clinical assessments and details of surgery (surgical lavage, material removal, one or two-stage revision), and antimicrobial therapy (empiric treatment defined as the antibiotics initiated in the period before the culture of surgical samples result, type of appropriate treatment defined as the antibiotics initiated according to the antimicrobial susceptibility profile after the culture results), duration of intravenous treatment and total duration of antibiotic treatment were collected. Early switch from intravenous to oral antibiotic therapy was defined as a switch in the first 5 days of antibiotic start. This cut-off was chosen because it reflected the mean length of stay in acute ward before transfer to other facilities (mainly rehabilitation center).

The primary outcome was treatment failure defined as the recurrence of *S. aureus* infection at any time during or after the first line of medical and surgical treatments within 2 years of follow-up. Secondary outcomes were the mean length of hospital stay, the prevalence of vascular catheter-related complications during the antibiotic treatment period and non-predefined factors associated with failure. Treatment failure did not include new episodes of infection on the same joint with microorganisms other than *S. aureus*.

## Statistical analysis

Results were expressed as absolute counts and percentages (n, %) for categorical variables and median with interquartile ranges (IQR) or mean with standard deviation (SD) for continuous variables. The median is presented in case of variables with skewed distribution.

Univariate comparisons were done using the Fisher’s exact tests for categorical variables and Student’s t-test for continuous variables. Cox regression model was used to identify independent risk factors for treatment failure. Variables found to be significant at the univariate levels (*p*-value ≤0.20) were included into the Cox model. Results were given as hazard ratio (HRs) with 95% confidence intervals (CIs).

A *p*-value < 0.05 was considered statistically significant. Data analyses were performed using SAS version 9.1 (SAS Institute Inc., Cary, NC, USA).

## Results

### Patients

**Fig. 1:**
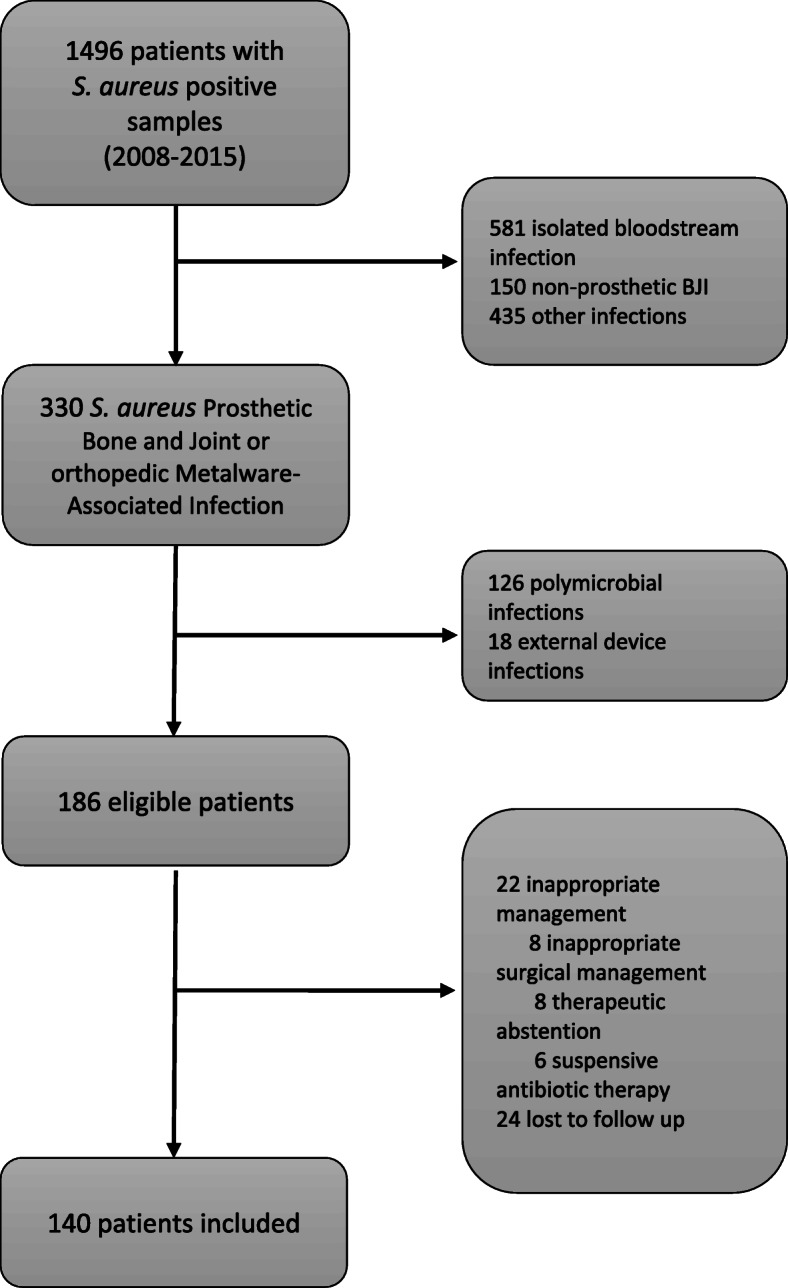
Flow chart of the study. BJI: Bone and joint infections

Of the 1496 patients identified with *S. aureus* infections during the inclusion period, 330 had PJI infections. One hundred forty-four patients were excluded for polymicrobial infection (*n* = 126) or infection of external devices (*n* = 18). Finally, 140 patients with *S. aureus* prosthetic bone and joint or orthopedic metalware-associated infection were included (Fig. 1). Mean age was 60.4 years (SD 20.2), and 66% were male (*n* = 92). The most frequent co-morbidities were diabetes mellitus (*n* = 21, 15%), chronic kidney disease (*n* = 12, 9%), cancer (*n* = 10, 7%), being treated with immunosuppressive therapy (*n* = 10, 7%), and antecedent radiotherapy therapy (*n* = 5, 4%). Mean body mass index (BMI) was 25.9 kg/m^2^ (SD 5.5) with 17% classified as obese (*n* = 24). Mean Charlson index was 2.11 (SD 1.98).

Fifty-three patients (38%) had a joint prosthesis including 38 total/intermediate hip replacement, 13 total knee replacement, 2 total ankle replacement and 87 (62%) had orthopedic material including 16 intramedullary nails and 71 plates screws and/or pins. Of note, 50 (36%) had undergone revision surgery prior to the septic episode because of mechanical failure of the prosthesis.

### Infections care

Infections were mainly due to methicillin-susceptible *S. aureus* (MSSA) (*n* = 113, 81%). The mean time from symptom onset to antibiotic initiation was 19.9 days (SD 22.5). The mean duration of intravenous and total antibiotic treatment was 4.1 days (SD 4.6) and 11.5 weeks (SD 4.5), respectively. The majority of patients (119, 85%) had 5 days or less of antibiotic intravenous therapy. Most oral antibiotic regimen included rifampicin (*n* = 118, 84%), combined with ofloxacin (*n* = 88, 63%) or sulfamethoxazole (*n* = 22, 16%). Of note, 24 patients (17%) had concomitant bacteremia of which 20 were considered as secondary bacteremia related to the prosthetic bone and joint or orthopedic metalware-associated infection and were treated with 5 days of less of intravenous antibiotic therapy before oral switching after exclusion of Infective Endocarditis by one (or two) transthoracic echocardiogram(s).

The median time from symptom onset to surgery was 9 days (IQR 4–23). Type of PJIs (according to the Coventry/Tsukayama classification [7, 8]) and surgical management according to type of prothesis/orthopedic material is detailed in Table 1.

**Table 1:** Type of prosthetic joint infection and surgical management according to type of prothesis/orthopedic material

	Total hip replacement	Total knee replacement	Total ankle replacement	Nail	Plate/Screw and/or pin
**Total**	38	13	2	16	71
**Male sex**	22 (58%)	8 (62%)	1 (50%)	8 (50%)	53 (75%)
**Mean age**, years (SD) {min - max}	74 (12.7) {39–96}	68 (14.7) {37–90}	54	60 (23.6) {21–89}	51.8 (19.5) {19–93}
**Charlson score** (SD) {min - max}	2.8 (2.2) {0–9}	2.5 (1.7) {0–6}	0.5 (0.7) {0–1}	2.5 (2.0) {0–6}	1.6 (1.9) {0–8}
**Methicillin susceptible** ***S. aureus*** (%)	28 (74%)	11 (85%)	2 (100%)	11 (69%)	61 (86%)
**Joint infection classification (Coventry1975) (Tsukuyama 1996)**					
Type I	0	0	0	2 (13%)	3
Type II	20 (53%)	1 (8%)	0	8 (50%)	58
Type III	13 (34%)	2 (15%)	0	6 (38%)	51
Type IV	5 (13%)	10 (63%)	2 (100%)	0	28
**Surgical management**					
Intra-articular wash^a^	11 (29%)	5 (39%)	1 (50%)	6 (38%)	30 (42%)
One-stage revision	4 (11%)	1 (8%)	0	4 (25%)	4 (6%)
Two-stage revision	12 (11%)	6 (46%)	0	0	3 (4%)
Material removal	0	0	0	6 (38%)	34 (48%)
Inserts change	11 (29%)	1 (8%)	1 (50%)	0	0
**Failure**	2 (5.3%)	5 (38.5%)	0	2 (12.5%)	3 (4%)

^a^open debridement without exchange of modular components

References: [7, 8]

### Treatment failure and associated risk factors

Twelve patients (8.5%) experienced treatment failure: four relapsed while under antibiotic therapy, three relapsed within the first month after antibiotic termination and the last five within 6 months after antibiotic termination.

In univariate analysis, methicillin-resistant *S. aureus* (MRSA) infections, infection of total knee replacement and type IV infection [8] were significantly associated with treatment failure (Table 2). In multivariate analysis, MRSA infections (aHR 11.1; 95% CI 1.5–111.1; *p* = 0.02), obesity (BMI > 30 kg/m^2^) (aHR 6.9; 95% CI 1.4–34.4, *p* = 0.02) and empiric antibiotic therapy not following the guidelines [4, 5] (aHR 7.1; 95% CI 1.8–25.2; *p* = 0.005) were significantly associated with treatment failure whereas a duration of less than 5 days of intravenous antibiotic therapy was not (aHR 0.8 95%CI 0.4–3.5, *p* = 0.66).

**Table 2:** Characteristics of patients and management according to the treatment outcome

	Failure n(%)	***p***-value
**Sex**		0.97
Male	8/92 (8.7)	
Female	4/48 (8.3)	
**Type of prosthetic material**		0.001
Total hip replacement	2/38 (5.3)	
Total knee replacement	5/13 (38.5)	
Total ankle replacement	0/2 (0)	
All prosthesis	7/53 (13.2)	
Intramedullary nailing	2/16 (12.5)	
Plate/screw/pin	3/71 (4.2)	
**Type of surgery**		0.33
Intra-articular wash^a^	6 (11.3)	
One-stage revision	2 (15.4)	
Two-stage revision	3 (14.3)	
Material removal	1 (2.5)	
Mobile inserts change	0 (0)	
**Type of** infection		0.01
Type I	0/5(0)	
Type II	2/58 (3.5)	
Type III	2/51 (4)	
Type IV	6/28 (21.5)	
Infection by inoculation	9/115 (7.8)	
**S. aureus susceptibility**		0.01
*Methicillin susceptible*	6/113 (5.3)	
*Methicillin resistant*	6/27 (22.2)	
**Intravenous antibiotic therapy**		0.15
Empiric therapy following guidelines	6/104 (5.7)	
Empiric therapy not following guidelines	6/26 (16.6)	
**Duration of intravenous antibiotic therapy**		0.47
≤ 5 days	9/119 (7.5)	
> 5 days	3/21 (14.2)	
**Oral antibiotic therapy**		0.42
Rifampicin + Ofloxacin	6/88 (6.8)	
Rifampicin + Cotrimoxazole	2/21 (4.8)	
Rifampicin + another antibiotic	2/8 (25.0)	
Combination without rifampicin	3/22 (13.6)	
**Chronic comorbidities**		0.17
Diabetes mellitus	2/21 (9.5)	
Obesity	4/24 (16.7)	
Chronic renal failure	1/12 (8.3)	
Cancer, history of radiotherapy, immunosuppressive therapy	0/25 (0)	

^a^open debridement without exchange of modular components

References [4, 5]

Overall, 6 patients (4%) died during the follow-up.

### Hospitalization duration and catheter-related infections

Mean length of stay was 14.4 days (SD 11.7). Patients with less than 5 days of intravenous antibiotic therapy had a significantly shorter length of stay (13.0 days (SD 10.4) vs 22.7 (SD 15.4) *p* = 0.015).

Only two patients (1%) presented catheter-related infections; both had had early switch from intravenous to oral antibiotic therapy (< 5 days).

## Discussion

The main objective of this study was to determine whether shorter postoperative intravenous antibiotic treatment in the management of *S. aureus* prosthetic bone and joint or orthopedic metalware-associated infection had an impact on treatment failure. In our cohort of 140 patients, the mean duration of intravenous treatment was 4 days, while current guidelines recommend a minimum of 14 days of parenteral treatment in such infections [4]. The overall treatment failure rate was 8.5%, without difference between short (≤ 5 days) and longer (> 5 days) intravenous antibiotic treatment duration. Finally, we found that MRSA, obesity and inappropriate empiric antibiotic therapy were independently associated with treatment failure.

The treatment failure rate found here is similar to that observed in the literature, which varies from 10 to 20% depending on the surgical treatment. A few retrospective studies have shown a high rate of success when doing early switching in different settings. In 2005, a cure rate of 87% was observed in a retrospective study of early acute infections of prostheses treated by lavage and combined rifampicin-ciprofloxacin with oral switch between 3 to 7 days [9]. In 2007, a 95% cure rate was reported in a single-center study of late and chronic total hip replacement infections treated by two-step replacement and early antibiotic switch at 5 days [10]. In 2011, a retrospective single-center study found a 89% cure rate on total hip replacement infections regardless of surgical treatment, with a median of 14 days for intravenous-to-oral antibiotic switch [11]. Finally, the recently published OVIVA study, a multicenter randomized controlled trial comparing 1 year outcomes between early (within 1 week) intravenous-to-oral antibiotic switch and continuation of intravenous therapy for at least 6 weeks in adults with bone and joint infections showed a high success rate (86%), irrespective of route of antibiotic administration [12]. In this study, two-thirds of patients had PJI and 40% of infections were due to *S. aureus*. Importantly, studies derived from the OVIVA trial have shown that early outpatient parenteral antimicrobial therapy could be used in 80% of patients [13] and would decrease length of stay, treatment costs and vascular device-related complications [12, 13].

Our results on the success rate and length of hospital stay are in line with the OVIVA study [12], however, contrary to the OVIVA study, we observed no difference in catheter-related complications between the two groups, probably because of the small size of the late switch group. Both patients in the early oral switch group who had a catheter-related infection had prolonged hospital stay and perfusion for other reasons than the prosthetic bone and joint or orthopedic metalware-associated infection. This highlights the frequent misuse of catheters, such as mid- or peripherally inserted central catheter-line, which are inserted assuming a prolonged intravenous therapy but used for other perfusions or maintained even after oral switch, increasing the risk of catheter-related infections.

Our results showing that MRSA was associated with treatment failure is controversial in the literature. Indeed, Volin et al. did not show any difference in the resistance profile of *S. aureus* compared to the success rate of PJI treatment on total knee replacement [14]. Conversely, Salgado et al. found a higher failure rate in PJI with MRSA than with MSSA [15]. Leung et al. also noted more failures in the management of total hip replacement infected with MRSA or methicillin-resistant *Staphylococcus epidermidis* compared to those infected with MSSA or methicillin-susceptible *S. epidermidis* [16]. Therefore, some authors have proposed establishing management recommendations including the nature of the microorganism involved [17]. Moreover, we recently observed a clear decrease of MRSA in Europe, which will have a wide-ranging positive effect on avoiding treatment failure [18].

This work had several limitations. First, this was a single-center study with a small sample size which may have jeopardize external validity of our results and have led to insufficient power to show any significant differences. Second, our center prioritizes early switching, meaning that the groups were not comparable for early/late switch. Furthermore, in this non-randomized retrospective study, patients with longer intravenous antibiotic therapy may have been selected because of more severe infection or less effective surgical treatment, which may have biased our results. Third, death was not considered as a competing risk for treatment failure, which may have led to underestimation of failure. Forth, the a priori choice of 5 day-cut-off for early/late switch can be discussed.

## Conclusion

This work adds to the growing body of evidence of high treatment success in early intravenous to oral switch antibiotic therapy in *S. aureus* prosthetic bone and joint or orthopedic metalware-associated infection. It is now important to clearly define the optimal oral antibiotic regimens and its duration for patient management, and to communicate the international guideline to avoid unnecessary use of intravenous therapy.

## Data Availability

The data that support the findings of this study are available on request from the corresponding author (PL). The data are not publicly available due to hem containing information that could compromise research participant privacy.
